# The Post-Resuscitative Urinalysis Associate the Survival of Patients with Non-Traumatic Out-of-Hospital Cardiac Arrest

**DOI:** 10.1371/journal.pone.0075172

**Published:** 2013-10-04

**Authors:** Chin-Fu Chang, Chao-Jui Li, Chih-Jan Ko, Tsung-Han Teng, Shih-Chang Lai, Mei-Chueh Yang, Chun-Wen Chiu, Chu-Chung Chou, Chih-Yu Chang, Yung-Chiao Yao, Lan-Hsin Wu, Han-Ping Wu, Wen-Liang Chen, Yan-Ren Lin

**Affiliations:** 1 Department of Emergency Medicine, Changhua Christian Hospital, Changhua, Taiwan; 2 Department of Emergency Medicine, Chang Gung Memorial Hospital - Kaohsiung Medical Center, Chang Gung University College of Medicine, Kaohsiung, Taiwan; 3 Department of General Surgery, Changhua Christian Hospital, Changhua, Taiwan; 4 Department of Biological Science and Technology, National Chiao Tung University, Hsinchu, Taiwan; 5 Department of Pathology, Taichung Hospital, Department of Health, Executive Yuan, Taichung, Taiwan; 6 School of Medicine, Chung Shan Medical University, Taichung, Taiwan; 7 Department of Nursing, Changhua Christian Hospital, Changhua, Taiwan; 8 Department of Pediatrics, Buddhist Tzu Chi General Hospital, Taichung Branch, Taichung, Taiwan; Gentofte University Hospital, Denmark

## Abstract

**Objective:**

To analyze whether urine output and urinalysis results are predictive of survival and neurologic outcomes in patients with non-traumatic out-of-hospital cardiac arrest (OHCA).

**Methods:**

Information was obtained from 1,340 patients with non-traumatic OHCA who had achieved a sustained return of spontaneous circulation (ROSC). Factors that were associated with survival in the post-resuscitative period were evaluated. The association between urine output and fluid challenge in the early resuscitative period was analyzed and compared between the survivors and the non-survivors. The results of the initial urinalysis, including the presence of proteinuria and other findings, were used to evaluate the severity of vascular protein leakage and survival. The association between proteinuria and the neurologic outcomes of the survivors was also analyzed. The clinical features of capillary leakage were examined during the post-resuscitative period.

**Results:**

Of the 1,340 patients, 312 survived. A greater urine output was associated with a higher chance of survival. The initial urine output increased in proportion to the amount of fluid that was administered during early resuscitation in the emergency department for the survivors but not for the non-survivors (p<0.05). In the initial urinalysis, proteinuria was strongly associated with survival, and severe proteinuria indicated significantly poorer neurologic outcomes (p<0.05 for both comparisons). Proteinuria was associated with a risk of developing signs of capillary leakage, including body mass index gain and pitting edema (both p<0.001).

**Conclusion:**

The severity of proteinuria during the early post-resuscitative period was predictive of survival.

## Introduction

The survival rate of patients with out-of-hospital cardiac arrest (OHCA) is low. [1]–[4] Although a sustained return of spontaneous circulation (ROSC) can be initially established following resuscitation from non-traumatic OHCA in certain patients, many of these patients lose spontaneous circulation during the hospital stay due to continued damage from post-cardiac arrest syndrome. [1], [3], [5]–[7].

The difficulties of prognostications in OHCA patients during the post-cardiac arrest period are well known. Systemic ischemia/reperfusion (I/R) injuries and related end-organ dysfunction contribute to death in the post-resuscitative period. [3], [7], [8] These injuries present immediately after achieving an ROSC in patients in the emergency department (ED), and the outcomes of these patients depend on several factors, including the timing of the treatment, as early hemodynamic optimization is associated with survival. [3], [9] Because a standard quantification procedure has not been developed for the severity of I/R injuries following OHCA, certain kidney-related biomarkers have been used, including serum creatinine, bicarbonate, and pH levels. [3], [10]–[12] However, these biomarkers are characterized by certain limitations. For example, a normal serum creatinine range may not indicate intact kidney function, especially during the early stage of an injury. For instance, a previous study reported that 51% of patients did not exhibit increased serum creatinine levels following cardiac arrest. [10] In addition, serum bicarbonate and pH levels may be influenced by various conditions (i.e., apnea-related CO_2_ retention, chronic lung diseases, and metabolic diseases). Previous studies have demonstrated that vascular protein leakage due to cell barrier dysfunction presents as soon as 30 min following shock and an I/R injury, [13]–[18] which is much earlier than myocardial dysfunction, which has been reported to occur at a median time of 6.8 h after OHCA. [6] Most importantly, in the kidney, glomerular barrier dysfunction and proteinuria were also demonstrated in patients with systemic I/R injuries. [13] Therefore, the presence of protein in the urine and symptoms of capillary leakage may be predictive of the severity of a systemic I/R injury in the post-resuscitative phase. However, the results of a urinalysis and the measurement of protein in the urine have never been analyzed as predictors of survival in OHCA patients. In the present study, we first hypothesized that the severity of an I/R injury might be reflected by vascular injury and ensuing protein leakage in OHCA patients. Additionally, we analyzed whether urine output and urinalysis results are predictive of survival during the early post-resuscitative period in patients with non-traumatic OHCA.

## Methods

### Study Design

**Figure 1:**
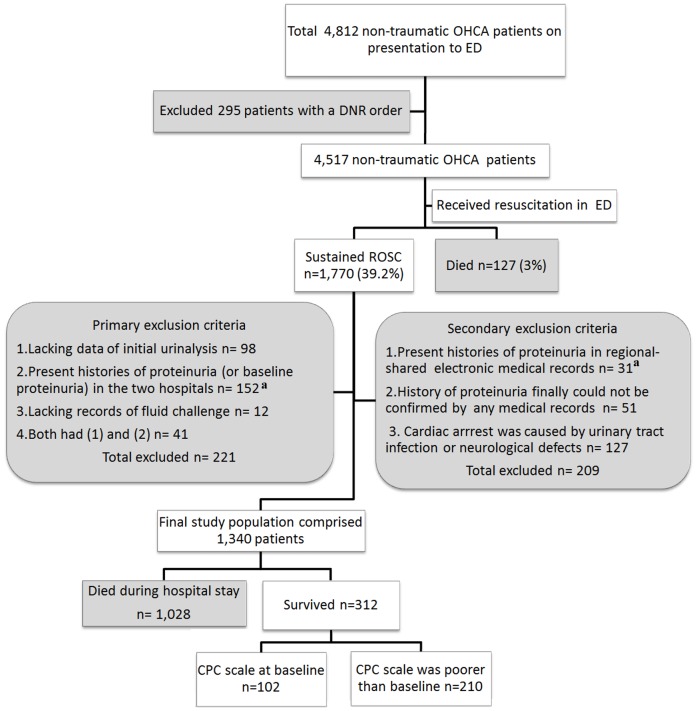
The selection of the participants and the primary outcomes of the patients. The baseline renal functions (proteinuria) of all patients were determined within 3 years before cardiac arrest. ^a^In total, 52 had diabetes-related proteinuria. OHCA: out-of-hospital cardiac arrest; ED: emergency department; DNR: do not resuscitate; ROSC: return of spontaneous circulation; CPC: cerebral performance categories.

During the period between January 1, 2007, and September 30, 2011, a total of 4,812 adult patients (>18 years of age) presented with non-traumatic OHCA at the ED of the Chang Gung Memorial Hospital (CGMH) or the Changhua Christian Hospital (CCH). A total of 1,770 patients who achieved a sustained ROSC following primary resuscitation in the ED were included in this retrospective study. The exclusion criteria are described in Figure 1. In this study, factors associated with the primary (survival to discharge) and secondary (neurologic outcomes) outcomes were analyzed.

### Ethics Statement

The study protocol was approved by the Institutional Review Board (IRB) of the Changhua Christian Hospital (IRB code: 121007). Consent was specifically waived by the approving IRB. To improve the accuracy of the findings and to minimize inconsistencies in the chart reviews, chart abstractors (ED physicians) were trained using a practice record prior to initiation of the study. The chart abstractors used a standardized abstraction form to guide the data collection, and the quality of their data was discussed at regular meetings. The performance of the chart abstractors was also monitored.

### Study Setting and Population

We retrospectively reviewed the medical records of 1,340 adult patients who presented with non-traumatic OHCA at the ED of the CGMH or the CCH. Clinically, the possible etiologies of the OHCA varied. In the present study, all of the patients were divided into nine groups based on the following possible etiologies: (1) idiopathic causes, (2) infection, (3) cardiovascular disease, (4) malignancy, (5) asphyxia due to acute or chronic respiratory failure, (6) electrolyte imbalance, (7) intoxication, and (9) hypovolemia. Unclear etiologies were classified as idiopathic. Neurologic defects or urinary tract infection was not included.

### Study Protocol

Information related to the pre-hospital phase of resuscitation, including the period during which the patient was treated at the scene and transported to the hospital and the patient’s possibly witnessed collapse, was obtained from the public emergency medical system records or witness statements. Information related to patient characteristics, the ED resuscitative phase, and the post-resuscitative phase was obtained from the medical chart records. The patients received in-hospital resuscitation according to advanced cardiopulmonary life support (ACLS), which is a standard American Heart Association protocol. The protocols followed the Utstein reporting system. [19] In the present study, in-hospital cardiopulmonary resuscitation (CPR) was defined as CPR that was performed in the ED, and the post-resuscitative phase was defined as the period after which the patients had achieved a sustained ROSC. The ED resuscitation phase was defined as the period of time beginning when the patients entered the ED and ending upon their arrival in the intensive care unit (ICU). The initial cardiac rhythm on presentation to the ED was obtained from electrocardiographic monitoring, which was immediately performed upon arrival. The rhythms included ventricular fibrillation (VF), pulseless ventricular tachycardia, pulseless electrical activity (PEA), and asystole. In the present study, a diagnosis of VF was also assigned to patients who exhibited pulseless ventricular tachycardia. The average duration of the ED resuscitation phase was 90 min. All of the decisions regarding treatments and the termination of resuscitation efforts were made at the discretion of the treating physician. The data that were gathered from the ED patient charts included age, sex, body mass index (BMI), the duration of in-hospital CPR, and fluid challenge during the ED resuscitation phase (including crystalloid fluid, colloid fluid, and blood products). Moreover, the total amount of fluid and the speed of challenge were decided by the ED treating physicians according to the clinical conditions of the patients. Data regarding the initial hemoglobin, potassium, pH, PaO2, PaCO2, lactic acid, and creatinine clearance levels were also obtained.

Urinary catheters were immediately inserted in all of the patients after they achieved a sustained ROSC. The urine that was drained from the urinary catheters during catheterization was regarded as residual urine and was not considered to represent post-resuscitative urine output. In the present study, the amount of post-resuscitative urine output was recorded, and the urine was routinely submitted for urinalysis. All of the urine samples were collected from the proximal urinary catheters via needle aspiration and aseptic manipulation within the first 6 hours of the hospital stay. The samples were immediately sent to the medical laboratories of the CGMH and the CCH. Therefore, the initial urinalysis was regarded as the initial post-resuscitative urinary analysis. Associations between the following urinary characteristics and survival were evaluated: specific gravity, the presence of proteinuria, the presence of pyuria, and the presence of hematuria. Urine measurements included urine chemistry (strip) and a microscopic examination. The results of urine chemistry (protein and gravity) were analyzed by a dipstick colorimetric test (Multistix Urine Strips, Bayer Diagnostics), which could be read visually by well-trained operators, requiring no additional equipment. However, the visibly bloody urines might cause falsely elevated proteinuria. Pyuria was defined as the presence of 10 or more neutrophils per high-power field. The volume of the administered fluid and the urine output in the early post-resuscitative period (i.e., the ED resuscitation phase) was compared with the same measures in the intermediate post-resuscitative period (i.e., the first 24 hours of the ICU stay). The total volume of the fluid challenge and the urine output during the two periods were compared between the survivors and the non-survivors.

The neurologic outcomes of the survivors were recorded according to the cerebral performance categories (CPC) scale prior to cardiac arrest (baseline) and at the time of discharge from the hospital. The baseline CPC scales of the survivors were also evaluated by their treating physicians based on the patient history that was provided by the patients’ family members. In the present study, the survivors were divided into two groups based on their neurologic outcomes: (1) CPC scale at baseline and (2) CPC scale lower than baseline. Factors associated with the neurologic outcomes of the survivors were analyzed. The relationships between the chance of survival, neurologic outcomes, and the severity of proteinuria were also analyzed.

The post-resuscitative I/R injuries that developed during the first 48 hours of the hospital stay may have presented with signs of capillary leakage, included the following: (1) a BMI increase (no increase, <0.5, 0.5–1, or >1 kg/m^2^) and (2) the presence of pitting edema. We also evaluated whether these complications and the severity of proteinuria (none, 0.01–1, 1.01–2, or >2 g/L) were associated with survival.

### Data Analysis

The descriptive analyses of the independent variables (patient characteristics, the possible etiologies of OHCA, pre-hospital resuscitation, ED resuscitation, post-resuscitative care, and the results of the urinalysis) are reported as percentages, the mean ± standard deviation (SD), or the median. The factors that may have been associated with survival to discharge and neurologic outcomes were analyzed using the Mann-Whitney U test, Fisher’s exact test, one-way ANOVA, or the Chi-squared test. The relationships between the magnitude of the fluid challenge and the urine output in the ED or during the first 24 hours of the ICU stay were analyzed using Spearman’s rank correlation test. The relationships between the severity of proteinuria (ordinal variable) and the outcomes of the survivors were analyzed using the Chi-squared test. The association between proteinuria and therapeutic hypothermia was analyzed by the Chi-squared test. Multivariate logistic regression analysis was used to determine the urinalysis-based metrics and the demographics of patients that most strongly predicted survival. Lastly, the most important clinical signs that could be associated with capillary leakage and that may reflect proteinuria severity were analyzed using multivariate logistic regression analysis. The variables with univariate comparisons with p<0.1 were subsequently included in the multivariate logistic regression analysis (backward selection). A p-value <0.05 was considered to be statistically significant. All of the analyses were performed using the statistical package SPSS for Windows (Version 15.0, SPSS Inc., Chicago, IL).

## Results

### The Characteristics of the Patients with a Sustained ROSC and the Factors that were Related to Survival

The patient characteristics are presented in Table 1. A total of 312 patients survived until discharge, and their neurologic outcomes at discharge are presented in Figure 1. Urine output was significantly higher in the patients who survived until discharge (1.2 ml/kg/h) than in the patients who died during the hospital stay (0.7 ml/kg/h) (p<0.001). Factors associated with survival and neurologic outcomes are also presented in Table 1.

**Table 1:** Factors associated with outcomes.

	Non-traumatic OHCA patients with a sustained ROSC (n = 1,340) No. (%)	Survival until discharge			Neurologic outcomes of survivors (n = 312)		
		Yes (n = 312) No. (%)	No (n = 1,028) No. (%)	p-value	CPC scale at discharge		
					CPC at baseline (n = 102)No. (%)	Poorer than baseline (n = 210)No. (%)	p-value
**Patient characteristics**							
Age (Mean ± SD) (y/o)	69.5±16.7	69.7±17.5	69.6±16.6	0.813	69.7±19.6	69.5±17.2	0.982
Body mass index (Mean ± SD) (kg/m^2^) (25)^a^	19.4±3.7	18.9±4.6	19.6±2.8	0.016	18.8±5.1	18.9±3.7	0.255
Male	782 (58.4)	197 (63.1)	575 (55.9)	0.017	65 (63.7)	132 (62.9)	0.525
Possible etiologies				<0.001			0.135
Idiopathic cause	38 (2.8)	7 (2.2)	31(3.0)		2 (2.0)	5 (2.4)	
Various infections	305 (22.8)	50 (16.0)	255 (24.8)		14 (13.7)	36 (17.1)	
Cardiovascular disease	435 (32.5)	133 (42.6)	302 (29.4)		44 (43.1)	89 (42.4)	
Malignancy	94 (7.0)	5 (1.6)	89 (8.7)		1 (1.0)	4 (2.0)	
Asphyxia	313 (23.4)	74 (23.7)	239 (23.2)		26 (25.5)	48 (22.9)	
Electrolyte imbalance	29 (2.2)	5 (1.6)	24 (2.3)		4 (3.9)	1 (0.5)	
Intoxication	53 (4.0)	17 (5.4)	36 (3.5)		3 (3.0)	14 (6.7)	
Hypovolemia	73 (5.4)	21 (6.7)	52 (5.1)		8 (7.8)	13 (6.2)	
**Pre-hospital resuscitative phase**							
Witnessed collapse	544 (40.6)	221 (70.8)	323 (31.4)	<0.001	97 (87.4)	105 (62.5)	<0.001
Period from scene to hospital (Mean ± SD) (min) (84)^a^	20.2±7.3	17.8±7.1	20.3±8.5	<0.001	17.1±6.2	18.1±5.4	0.391
**ED resuscitative phase**							
Initial cardiac rhythm				<0.001			<0.001
Asystole	877 (65.4)	137 (43.9)	740 (72.0)		29 (28.4)	108 (51.5)	
VF^b^	316 (23.6)	121 (38.8)	195 (19.0)		59 (57.8)	62 (29.5)	
PEA	147 (11.0)	54 (17.3)	93 (9.0)		14 (13.7)	40 (19.0)	
Duration of in-hospital CPR (Mean ± SD) (min)	12.1±7.8	11.9±9.2	12.4±7.6	0.323	10.5±7.7	12.9±9.6	0.231
**Post-resuscitative phase**							
Therapeutic hypothermia	156 (11.6)	48 (15.4)	108 (10.5)	<0.001	22 (21.6)	26 (12.4)	<0.001
Urine output in first 24 hours of ICU stay (Median)(ml/kg/h) (30)^a^	0.9	1.2	0.7	<0.001	1.2	1.0	0.039
Initial hemoglobin level (Median) (mmol/L)	7.2	7.8	6.6	<0.001	8.1	7.8	0.844
Initial K level (Median) (mmol/L)	4.6	4.3	4.9	<0.001	3.9	4.4	0.972
Initial pH level (Median)	7.07	7.13	7.05	<0.001	7.25	7.14	0.224
Initial PaO2 level (Median) (mmHg)	156.8	183.9	139.4	<0.001	165.3	181.3	0.950
Initial PaCO2 level (Median) (mmHg)	58.5	47.2	59.1	<0.001	47.4	62.5	0.039
Initial creatinine clearance (Median) (mL/s)	1.1	1.3	0.8	<0.001	1.6	1.2	0.041
Initial lactic acid level (Median) (mmol/L) (29)^a^	2.6	2.1	3.5	<0.001	1.7	2.2	0.007

The factors associated with survival and neurologic outcomes in the patients who achieved a sustained ROSC following ED resuscitation.

CPC: cerebral performance categories; ICU: intensive care unit; VF: ventricular fibrillation; PEA: pulseless ventricular tachycardia; ROSC: return of spontaneous circulation; OHCA: out-of-hospital cardiac arrest; ED: emergency department.

a The number of patients with missing information.

b VF includes patients with pulseless VT.

### The Survival Likelihood Based on the Results of the Urinalysis

The majority of the patients presented with proteinuria (70.4%) or hematuria (65%). The predictive factors that were associated with survival and neurologic outcomes are given in Table 2. Moreover, Table 3 shows the variables (after adjusting for the contents of the urine and the demographics of the patients) that were associated with survival to discharge and neurologic outcomes.

**Table 2:** The association between urinalysis and survival.

	Non-traumatic OHCA patients with a sustained ROSC(n = 1,340) No. (%)	Survival until discharge			Neurologic outcomes of survivors (n = 312)		
		Yes (n = 312) No. (%)	No (n = 1,028) No. (%)	*p*-value	CPC scale at discharge		
					CPC at baseline (n = 102)No. (%)	Poorer than baseline (n = 210)No. (%)	p-value
**Gravity of urine**	1.016±0.012	1.022±0.022	1.015±0.017	<0.001	1.025±0.026	1.022±0.019	0.821
**Proteinuria**	944 (70.4)	143 (45.8)	801 (77.9)	<0.001	36 (35.3)	107 (51.0)	<0.001
**Pyuria**	341 (25.4)	65 (20.8)	276 (26.8)	<0.001	34 (33.3)	31 (14.8)	0.124
**Hematuria**	871 (65.0)	168 (53.8)	703 (68.4)	<0.001	53 (52.0)	115 (54.8)	0.534

The association between the results of the urinalysis (performed in the ED) and survival.

OHCA: out-of-hospital cardiac arrest; CPC: cerebral performance categories; ROSC: return of spontaneous circulation.

**Table 3:** Adjusting variables for outcomes.

	Survival until discharge			CPC scale at baseline when discharged from hospital^a^		
	OR	95% CI		OR	95% CI	
		Lower	Upper		Lower	Upper
**Patient characteristics**						
Body mass index^b^	1.04	1.01	1.07	0.93	0.41	1.92
Sex						
Female	0.6	0.34	1.11	0.7	0.22	3.73
Male	1			1		
Possible etiologies						
Idiopathic cause	2.02	0.51	8.01	4.11	0.69	5.12
Various infections	1.33	0.61	2.92	1.51	0.80	3.12
Cardiovascular diseaseb,c	27.56	7.46	101.96	18.10	5.21	73.42
Malignancy	2.01	0.91	4.46	3.11	0.26	7.53
Asphyxia	0.86	0.14	5.21	1.71	0.24	6.54
Electrolyte imbalance^b^	0.17	0.05	0.58	0.91	0.19	2.58
Intoxication^b^	0.14	0.04	0.49	0.25	0.16	1.73
Hypovolemia	1			1		
**Pre-hospital resuscitative phase**						
Witnessed collapse						
Yesb,c	3.21	2.42	7.53	4.16	3.40	11.71
No	1			1		
Period from scene to hospital^c^	1.01	0.96	1.06	0.85	1.12	5.32
**ED resuscitative phase**						
Initial cardiac rhythm						
VFb,c	7.82	1.22	9.43	5.12	3.21	10.10
PEAb,c	1.66	2.63	5.47	2.33	2.16	5.82
Asystole	1			1		
Therapeutic hypothermia						
Yesb,c	12.79	2.84	11.21	13.52	1.25	8.53
No	1			1		
**Post-resuscitative phase**						
Urine output in first 24 hours of ICU stayb,c	1.56	1.19	2.04	1.89	2.57	9.55
Initial hemoglobin level^b^	0.62	0.53	0.72	0.82	0.50	5.79
Initial K level^b^	1.46	1.12	1.90	1.45	0.74	9.12
Initial pH level^b^	0.01	0.01	0.07	0.10	0.03	2.54
Initial PaO2 level^b^	0.99	0.98	0.99	1.03	0.54	1.29
Initial PaCO2 level^b^	0.97	0.95	0.98	1.27	0.88	3.57
Initial creatinine clearanceb,c	1.63	1.12	2.37	2.54	2.74	5.40
Initial lactic acid levelb,c	2.52	2.74	9.10	3.56	1.09	6.21
**Urinalysis**						
Gravity of urine	0.72	0.72	2.11	0.52	0.87	4.20
Proteinuria						
Nob,c	1.94	1.34	2.52	6.58	1.92	12.52
Yes	1			1		
Pyuria						
No	0.85	0.45	1.61	1.63	0.35	3.24
Yes	1			1		
Hematuria						
No^b^	0.17	0.10	0.33	0.42	0.71	1.58
Yes	1			1		

The adjusting variables that were associated with survival to discharge and neurologic outcomes.

CPC: cerebral performance categories; ICU: intensive care unit; VF: ventricular fibrillation; PEA: pulseless ventricular tachycardia; OR: odds ratio; CI: confidence interval; ED: emergency department.

a For survivors.

b Significant factors associated with survival.

c Significant factors associated with better neurologic outcome.

### The Relationship between the Volume of Fluid that was Administered and Urine Output in the Patients with a Sustained ROSC

#### An early balance during ED resuscitation was associated with survival

**Figure 2:**
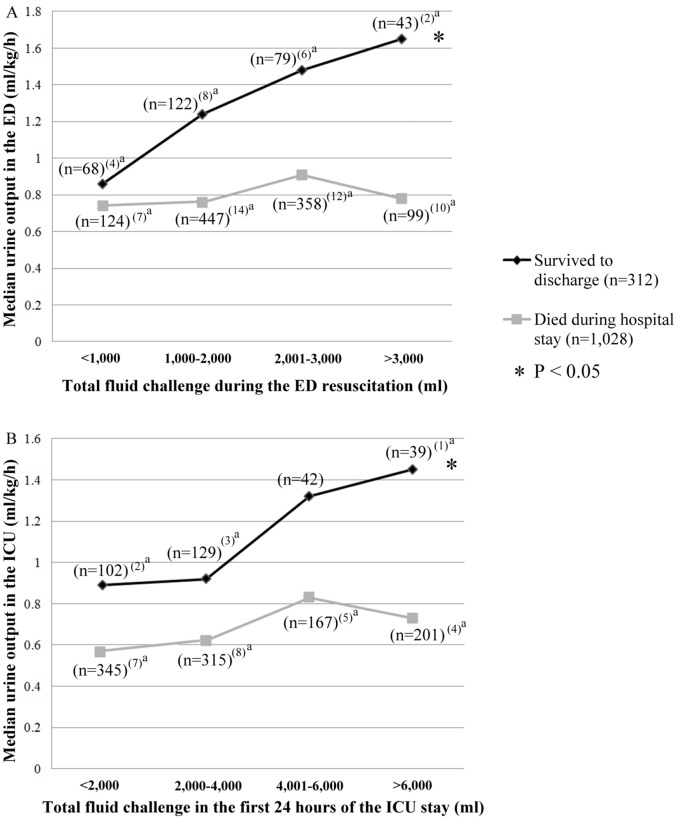
Urine output is associated with survival. The urine output of the patients with a sustained ROSC (A) in the ED and (B) during the first 24 hours of the ICU stay (Spearman’s rank correlation test). Urine output was significantly increased in proportion to the magnitude of the fluid challenge (A) during ED resuscitation (p<0.001, r = 0.86) and (B) in the first 24 hours of the ICU stay in the patients who survived until discharge (p = 0.002, r = 0.81). However, in the patients who died during the hospital stay, (A) urine output did not significantly increase in proportion to the amount of fluid that was administered during the initial ED resuscitation period (B) but did increase during the first 24 hours of the ICU stay when the total fluid administered was ≤6000 ml. ^a^The number of patients with missing urine output information.

The mean early post-resuscitative phase, which was defined as the period beginning when a sustained ROSC was achieved and ending upon admission to the ICU, was 90 minutes. During this period, the patients who survived until discharge exhibited an increase in initial urine output, and this output was proportional to the amount of fluid that was administered during ED resuscitation. This result indicates that an early establishment of hemodynamic balance may be associated with survival (p<0.05, r = 0.86) (Figure 2A).

#### Non-survivors presented with lower urine output in the first 24 hours of the ICU stay

During the ICU stay, hemodynamic balance was also obviously associated with survival (p<0.05, r = 0.81) (Figure 2B). There were six survivors who underwent posterior dialysis after discharge.

### The Severity of Proteinuria was Associated with Survival and Neurologic Outcomes

**Figure 3:**
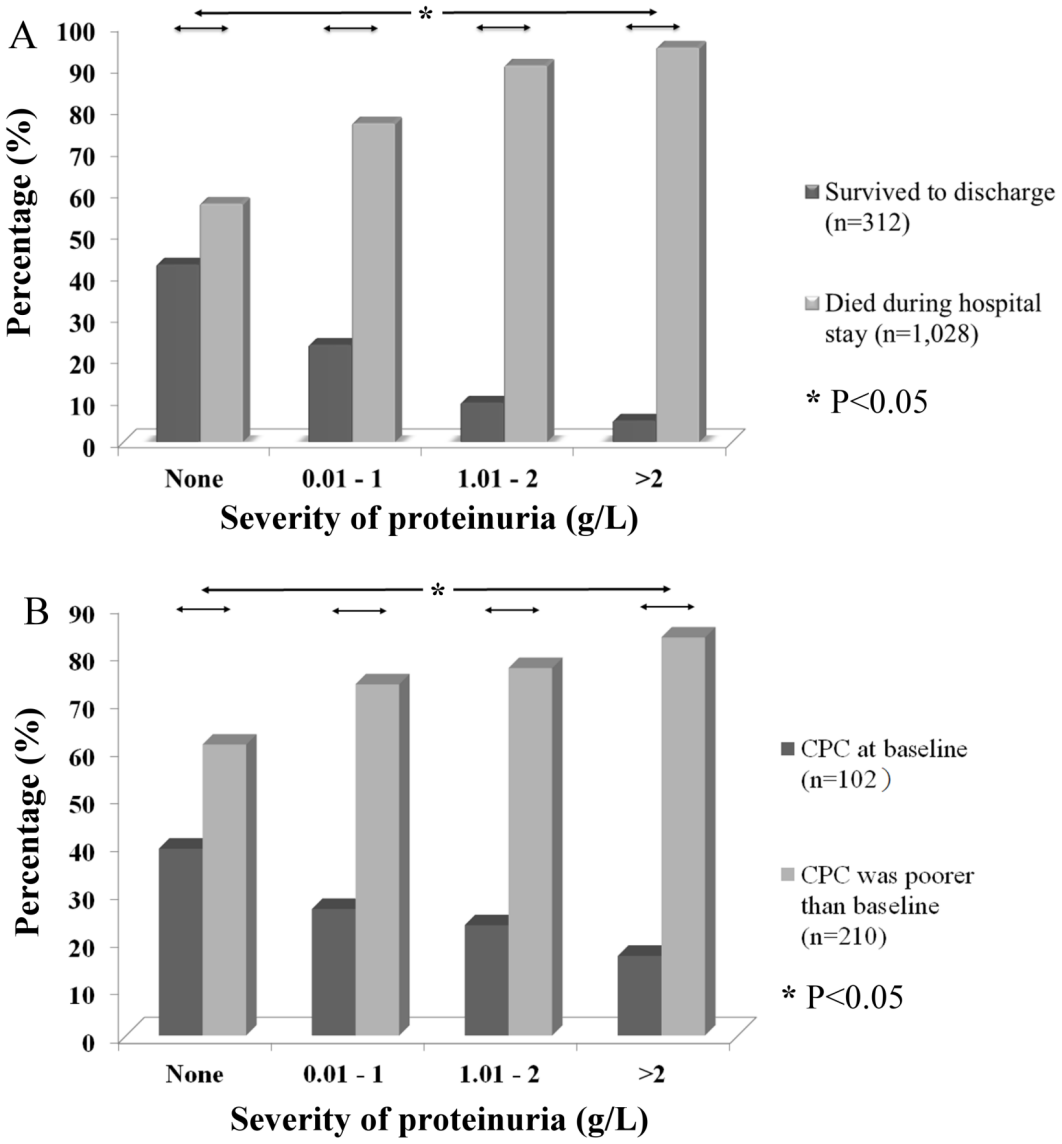
The severity of proteinuria is associated with outcomes. The severity of proteinuria was significantly associated with (A) survival and (B) neurologic outcomes at the time of discharge (Chi-squared test). More severe proteinuria might indicate significantly poorer neurologic outcomes in survivors.

Proteinuria was less common in patients who received therapeutic hypothermia (31 of 156; 19.9%) than in those patients who did not receive this treatment (913 of 1,184; 77.1%) (p<0.001). Among the 312 patients who survived until discharge, 54.2% did not exhibit proteinuria based on the initial urinalysis. More severe proteinuria indicated a significantly lower percentage of survivors (p<0.05) (Figure 3A). Furthermore, we noted that the severity of initial proteinuria was associated with the neurologic outcomes of the survivors. Among the survivors who did not show initial proteinuria, 39.1% exhibited a better neurologic outcome (CPC scale at baseline). However, among the survivors who had proteinuria that was higher than 2 g/L in their initial urine output, 83.3% presented with a poorer neurologic outcome (p<0.05) (Figure 3B).

### Other Factors that were Associated with Capillary Leakage were also Associated with Survival

Signs of capillary leakage that were present during the first 48 hours of the hospital stay in patients with severe proteinuria were associated with a decreased chance of survival (Table 4). After analyzing two clinical signs (BMI increase and pitting edema) in a regression model, pitting edema was found to be the symptom that was most significantly associated with different proteinuria severities (proteinuria range 0.01–1 g/L, OR: 3.51, 95% CI: 3.12–10.21; proteinuria range 1.01–2 g/L, OR: 4.53, 95% CI: 2.17–6.44; proteinuria range >2 g/L, OR: 12.3, 95% CI: 11.5–98.14; all p<0.001).

**Table 4:** Capillary leakage and survival.

	Non-traumatic OHCA patients with a sustained ROSC (n = 1,340)							
	No. who presented with proteinuria in the initial urinalysis (g/L)					Survival until discharge		
	None (n = 396)	0.01–1 (n = 418)	1.01–2 (n = 411)	>2 (n = 115)	p-value	Yes	No	p-value
	No. (%)	No. (%)	No. (%)	No. (%)		No. (%)	No. (%)	
**Body mass index increase (kg/m^2^) (279)** a					<0.001			<0.001
No increase	137 (38.3)	82 (25.6)	41 (14.1)	10 (10.8)		102 (36.6)	152 (19.4)	
<0.5	132 (36.9)	128 (40.0)	39 (13.4)	8 (8.5)		114 (40.9)	186 (23.8)	
0.5–1	37 (10.3)	63 (19.7)	83 (28.6)	10 (10.8)		36 (12.9)	161 (20.6)	
>1	52 (14.5)	47 (14.7)	127 (43.8)	65 (69.9)		27 (9.7)	283 (36.2)	
**Pitting edema (43)** a	76 (19.8)	258 (62.8)	356 (87.9)	93 (95.9)	<0.001	128 (43.4)	661 (65.9)	<0.001

The new-onset clinical presentations during the first 48 hours of the hospital stay that may have been associated with capillary leakage and survival.

a The number of patients with missing information.

## Discussion

Patients with OHCA who survive during the post-resuscitative period are at risk of developing a “sepsis-like” syndrome. [20], [21] Due to similarities between the post-cardiac arrest state and sepsis, it has been postulated that early goal-directed hemodynamic optimization and strategies that aim to maintain a urine output above 0.5 ml/kg/h may improve the outcomes of OHCA survivors. [3], [22] The early optimal urine output level during the ED resuscitative phase in patients with non-traumatic OHCA has not been established. We observed (i) that most OHCA survivors exhibited a median urine output >0.9 ml/kg/h during the ED resuscitative phase and (ii) that increased urine output indicated a higher probability of survival. Laurent et al. reported that an adequate fluid challenge led to a decrease in the severity of myocardial dysfunction in the post-resuscitative period. [6] In the present study, 122 survivors and 457 non-survivors each received more than 2,000 ml of a fluid challenge in the ED. The median urine output in the ED was significantly higher in the survivors than in the non-survivors. Similar patterns were noted when the administered fluid volume was either 2,001–3,000 ml or >3,000 ml. We therefore suspect that the stronger correlation that was observed between fluid input and urine output (indicating an early establishment of hemodynamic balance) was more important to survival than a single, massive fluid challenge.

Clinical evidence of an I/R injury in OHCA patients is often limited. A previous study reported that I/R injuries are a major cause of death in patients with OHCA during the post-resuscitative period. [3] Certain studies have provided evidence that cell barrier dysfunction and vascular hyperpermeability can be caused by an I/R injury via a local inflammatory response, resulting in vascular protein leakage. Glomerular barrier dysfunction and proteinuria have also been observed in the kidneys of patients with systemic I/R injuries.[13]–[18], [23] Hiratsuka et al. reported that these responses could be observed via microscopy within 30 min of an I/R injury in rats. [18] We therefore suspected that proteinuria may be the initial clinical presentation of a post-resuscitative I/R injury. Proteinuria would present much earlier than myocardial dysfunction, which has been reported to occur at a median time of 6.8 h following OHCA. [6] However, the importance of proteinuria during the post-resuscitative period has never been discussed in the context of patients with OHCA. We observed that proteinuria was predictive of survival in patients with OHCA. Severe proteinuria may reflect cell barrier dysfunction and indicate a complete or long-lasting, ongoing I/R injury. It has been reported that post-cardiac-arrest brain injury is caused not only by initial ischemic/hypoxic injuries but also by subsequent systemic inflammatory reactions. [3], [24] We suspect that this pattern of brain injury may be similar to that of post-cardiac-arrest kidney injury. Therefore, I/R injury-induced proteinuria and the local inflammatory reaction in the glomeruli might predict neurologic outcomes in OHCA survivors.

Capillary leakage can also be caused by severe cell barrier dysfunction. Therefore, capillary leakage may be systemic and could result in several complications in OHCA patients with a post-resuscitative I/R injury. Although the results of an organ biopsy can reveal capillary leakage, a biopsy may not be performed in every patient with an ROSC. In the present study, we demonstrated that the presentations of capillary leakage were significantly associated with the severity of proteinuria. Most importantly, early pitting edema was strongly associated with the presence of proteinuria and may further indicate the presence of capillary leakage and a systemic I/R injury. Overall, the clinical presentations of capillary leakage combined with initial proteinuria were indicative of poor outcomes in patients with non-traumatic OHCA.

### Limitations

This aim of this study was not to replace the well-established biomarkers (serum creatinine, lactic acid, bicarbonate, pH, glucose, and even urinary glomerular filtration) that have been reported to be associated with the outcomes of patients with cardiac arrest. [2], [3], [7], [10], [12], [25] Moreover, certain clinical biomarkers, such as iohexol clearance, cystatin C, and β-trace protein, have been demonstrated to reflect kidney functions early. The determination of the plasma clearance of iohexol is a simple, rapid, and accurate method that can be used to estimate the glomerular filtration rate (GFR) in critical patients. [26] Cystatin C has a low molecular weight (approximately 13.3 kilodaltons) and is removed from the bloodstream by glomerular filtration in the kidneys. Serum levels of cystatin C are a more precise test of kidney function (as represented by the GFR) than serum creatinine levels. [27], [28] β-trace protein has also emerged as a promising, novel endogenous marker for the GFR, representing a more sensitive marker for mild kidney dysfunction than serum creatinine. [29], [30] However, our results provide evidence (i) that proteinuria could be present in the early post-resuscitative period and (ii) that proteinuria is associated with certain symptoms of capillary leakage and with patient outcomes. The major limitation of this retrospective study was the determination of the histories of proteinuria (or baseline proteinuria levels). This limitation was unavoidable given that the occurrence of a cardiac arrest is typically unexpected. Therefore, evidence of new-onset proteinuria before cardiac arrest was typically unavailable. Although we carefully surveyed the past medical records of all of the patients, we suspect that a subset of the patients may have developed proteinuria during the period between a negative proteinuria finding (from a previous urinalysis) and cardiac arrest. According to our primary results, the different severities of proteinuria were also associated with different outcomes. Therefore, we suspect that this parameter may be useful in the prediction of outcomes in patients with baseline proteinuria or in those patients who present with comorbid conditions that contribute to proteinuria. The analysis of the relationship between fluid input and urine output also had certain limitations. The treatment strategies for fluid administration did not follow the same protocol, and the addition of a control group that received no fluid during the resuscitation was not feasible. Moreover, the treatment strategies of the different treating physicians varied according to the clinical conditions. The results of this study support the inference that an early establishment of hemodynamic balance is associated with survival. However, the direction of causation needs to be confirmed, as the survivors may have exhibited better urine output because of less damage and better circulation. There was a large amount of missing data for certain signs of capillary leakage (Table 4), given (i) that many patients died within the first 48 hours of the hospital stay and (ii) that the new-onset symptoms were not easily noted. Lastly, the physiologic explanations for the observed correlations may be oversimplified, and there may have been more contributing factors than those that were controlled for in this unadjusted model. This topic warrants further analysis in animal studies.

## Conclusion

The results of early urinalyses indicated initial systemic, I/R injury-induced vascular protein leakage. Most importantly, the severity of initial proteinuria predicted both survival and neurologic outcomes in the post-resuscitative period.
